# Acinetobacter lwoffii Cellulitis in an Immunocompromised Patient With Decompensated Cirrhosis, Diabetes Mellitus, and Psoriasis Vulgaris: A Case Report

**DOI:** 10.7759/cureus.97617

**Published:** 2025-11-23

**Authors:** Minoru Sakakiyama, Koji Hayashi, Hidetaka Matsuda, Maho Hayashi

**Affiliations:** 1 Department of Internal Medicine, Fukui General Hospital, Fukui, JPN; 2 Department of Endocrinology, University of Fukui, Fukui, JPN; 3 Department of Rehabilitation Medicine, Fukui General Hospital, Fukui, JPN; 4 Department of Gastroenterology, Fukui General Hospital, Fukui, JPN

**Keywords:** acinetobacter spp, acinetobacter wolffii, cirrhosis, diabetes mellitus, immunocompromised patient, metabolic dysfunction-associated steatotic liver disease (masld), psoriasis vulgaris, type 2 diabetes mellitus (dm)

## Abstract

This report describes a case of *Acinetobacter lwoffii* cellulitis in a 68-year-old woman with multiple immunocompromising conditions. The patient had a history of decompensated cirrhosis secondary to metabolic dysfunction-associated steatotic liver disease (MASLD), type 2 diabetes mellitus, and chronic plaque psoriasis. She was initially admitted with left lower limb pain and subsequently developed cellulitis that responded transiently to cefazolin therapy. Three months after discharge, she was readmitted for a superficial skin defect of the left leg and later developed right thigh cellulitis during cefazolin prophylaxis. Blood cultures grew *A. lwoffii*, which was susceptible to levofloxacin. The patient was successfully treated with levofloxacin and intravenous immunoglobulin, with resolution of fever and improvement of local inflammatory signs. This case highlights the clinical significance of *A. lwoffii*, an organism of relatively low virulence, as a causative pathogen in patients with complex immunocompromised states. The convergence of decompensated cirrhosis, diabetes, and psoriasis likely predisposed the patient to this rare infection. Clinicians should be aware of the potential for unusual gram-negative organisms to cause cellulitis in severely immunocompromised individuals and consider culture-directed antimicrobial therapy in management.

## Introduction

Cellulitis is a common bacterial skin and soft tissue infection that can affect both immunocompetent and immunocompromised individuals; however, immunocompromised patients demonstrate increased susceptibility, higher risk for atypical pathogens, and more severe clinical courses [1,2]. In the general population, cellulitis is primarily caused by gram-positive organisms, particularly *Streptococcus pyogenes* and *Staphylococcus aureus* [1,2]. However, in immunocompromised populations, the causative pathogen spectrum shifts significantly, with gram-negative bacteria accounting for up to 33% of cases in some series [3]. Understanding this epidemiologic shift is critical for appropriate empiric antimicrobial selection.

Liver cirrhosis represents a particularly high-risk state for skin and soft tissue infections. Population-based studies have demonstrated that cirrhotic patients have a 1.66-fold increased risk of developing cellulitis compared to non-cirrhotic controls (adjusted hazard ratio 1.66, 95%CI 1.52-1.82) in a Taiwanese national cohort of over 40,000 patients [4]. The immunodeficient state in cirrhosis is multifactorial, resulting from complement deficiency, impaired neutrophil chemotaxis and phagocytosis, reduced opsonization capacity, and altered gut barrier function leading to bacterial translocation [5]. Gram-negative organisms, particularly Enterobacteriaceae (*Escherichia coli*, *Klebsiella* spp.) and non-fermenters (*Pseudomonas aeruginosa*, *Acinetobacter* spp.), are increasingly recognized as causative pathogens in cirrhotic patients with cellulitis, with mortality rates reaching 30-56% in some series when bacteremia is present [6].

Diabetes mellitus represents another major independent risk factor for skin and soft tissue infections. Diabetic patients demonstrate impaired neutrophil function (chemotaxis, phagocytosis, and intracellular killing), compromised cell-mediated immunity, and microangiopathy leading to reduced tissue perfusion [7,8]. Hyperglycemia directly impairs immune cell function and promotes bacterial adherence to epithelial surfaces, creating an environment conducive to both typical and atypical bacterial invasion. Studies have shown that diabetic patients with cellulitis are more likely to have polymicrobial infections and gram-negative involvement compared to non-diabetic patients [7].

Psoriasis vulgaris, a chronic inflammatory skin disorder, may further compromise both local skin barrier function and systemic immunity. A recent hospital-based study of 200 psoriasis patients found that 21.5% experienced bacterial infections during hospitalization, with cellulitis ranking among the most common presentations [9]. The chronic systemic inflammation characteristic of psoriasis is associated with dysregulated immune responses, and immunosuppressive treatments commonly used for moderate-to-severe disease (including biologics, methotrexate, and cyclosporine) further increase infection risk [10]. Importantly, even without systemic immunosuppressive therapy, the impaired epidermal barrier and local immune dysfunction in psoriatic plaques may serve as potential portals for bacterial entry.

The convergence of multiple immunocompromising conditions, including decompensated cirrhosis, diabetes mellitus, and psoriasis, creates a synergistic and profound state of immune dysregulation. Shared mechanisms, including neutrophil dysfunction, complement deficiency, and compromised microvascular circulation, act cumulatively to increase susceptibility to opportunistic and atypical pathogens. In such complex immunodeficient states, even organisms of low virulence may cause significant invasive infections [11].

*Acinetobacter lwoffii *(formerly *Acinetobacter calcoaceticus var. lwoffii*) is a gram-negative coccobacillus recognized as normal flora of the skin, oropharynx, and perineum in healthy individuals [12]. Unlike the highly pathogenic *Acinetobacter baumannii*, which is a notorious cause of nosocomial infections and demonstrates extensive antimicrobial resistance, *A. lwoffii *is generally considered to have low pathogenic potential [12,13]. However, it can cause opportunistic infections in severely immunocompromised hosts, most commonly catheter-related bacteremia [13]. Cellulitis caused by *A. lwoffii *is exceedingly rare, with fewer than 10 cases reported in the literature, predominantly in patients with hematologic malignancies or end-stage renal disease on dialysis. Recent surveillance data indicate that *A. lwoffii* maintains relatively favorable antimicrobial susceptibility profiles compared to *A. baumannii*, with most isolates remaining susceptible to fluoroquinolones, carbapenems, and trimethoprim-sulfamethoxazole, though intrinsic resistance to first-generation cephalosporins and aminopenicillins is well-documented due to chromosomal β-lactamase production [13,14].

We present what appears to be the first reported case of *A. lwoffii* cellulitis in a patient with the combined triad of decompensated cirrhosis, diabetes mellitus, and psoriasis vulgaris. This unique convergence of immune-dysregulating conditions and barrier dysfunction provides important insights into the potential for typically low-virulence organisms to cause invasive disease in complex immunocompromised hosts. This case underscores the importance of considering atypical gram-negative pathogens in severely immunodeficient patients and highlights the value of culture-directed antimicrobial therapy.

## Case presentation

Patient background and first hospitalization

A 68-year-old woman presented to the emergency department with left lower limb pain and numbness and was admitted to our hospital. Her medical history was significant for multiple chronic immunocompromising conditions: decompensated liver cirrhosis secondary to metabolic dysfunction-associated steatotic liver disease (MASLD), which developed in her 40s, type 2 diabetes mellitus, diagnosed at age 40, and chronic plaque psoriasis, present since her 30s. Additionally, she had hyperlipidemia.

Her diabetes was managed with oral sitagliptin monotherapy, maintaining an HbA1c of 6-7%. She had a 30-year history of psoriasis treated with oral etretinate and topical calcipotriol; biologic therapy had never been administered due to concerns regarding infection risk in the setting of advanced liver disease. Despite treatment, her skin condition remained suboptimally controlled, with multiple erythematous, scaly plaques approximately the size of fingernail beds scattered over both lower legs, some with overlying fissuring. Around age 65, progressive hepatic fibrosis led to decompensated cirrhosis manifesting as hepatic encephalopathy, severe hypoalbuminemia (2.0-3.0 g/dL; reference range: 4.1-5.1 g/dL), and massive ascites, corresponding to Child-Pugh class C (score 10) [15]. No liver biopsy had been performed; the diagnosis of MASLD was based on clinical, laboratory, and imaging findings in the absence of other etiologies.

On admission, physical examination revealed marked bilateral lower extremity edema extending to the thighs, but no local signs of inflammation (erythema, warmth, or tenderness) were present at that time. Initial blood tests showed only mild elevation of C-reactive protein (CRP) at 3.40 mg/dL (reference range: 0.00-0.14 mg/dL), with white blood cell count within normal limits. Therefore, antibiotics were not initiated, and management focused on ascites control with diuretic adjustment and hepatic encephalopathy treatment.

On hospital day 11, the patient developed acute fever (38.5℃) accompanied by sharply demarcated erythema, marked warmth, edema, and tenderness of the left lower leg. The affected area measured approximately 15 cm in diameter and did not involve pre-existing psoriatic plaques. Laboratory evaluation revealed leukocytosis (16,800/μL; reference range: 3,300-8,600/μL) with neutrophil predominance (89.9%; reference range: 40.0-75.0%) and elevated CRP (7.99 mg/dL). Two sets of blood cultures were obtained and subsequently returned negative. Based on clinical presentation, she was diagnosed with left lower limb cellulitis, and empiric therapy with intravenous cefazolin (CEZ) 4 g/day (2 g every 12 hours) was initiated, targeting typical gram-positive pathogens.

The patient defervesced within 48 hours of CEZ initiation, and inflammatory markers improved (CRP decreased to 3.21 mg/dL by day 18). Local erythema and warmth also gradually diminished. Given the clinical improvement, CEZ was dose-reduced to 2 g/day on day 18 as step-down therapy.

However, on day 23, although the local inflammatory signs in the left lower leg remained stable without visible progression, serum inflammatory markers rose again (CRP 8.09 mg/dL, WBC 12,400/μL), raising concern for incomplete resolution or subclinical progression. CEZ was increased back to 4 g/day. Over the subsequent weeks, the patient experienced intermittent low-grade fevers (37.5-38.0°C) without clear identification of an alternative infection source despite evaluation for spontaneous bacterial peritonitis (negative paracentesis) and aspiration pneumonia (negative chest imaging). Given the patient's profound immunocompromised state (Child-Pugh C cirrhosis with albumin 2.3 g/dL) and slow clinical response, CEZ was continued with gradual improvement of inflammatory markers. After confirmation of sustained defervescence and CRP normalization (1.2 mg/dL), CEZ was discontinued on day 45 (total duration: 34 days). We acknowledge that this prolonged treatment course exceeded typical cellulitis management guidelines (five to seven days)[16]; the extended duration reflected the complexity of the patient's immunodeficient state, recurrent fever, and diagnostic uncertainty regarding alternative infection sources. The patient's ascites was controlled through diuretic optimization, and she was discharged on day 68 with planned outpatient follow-up.

Second hospitalization (three months later)

**Figure 1:**
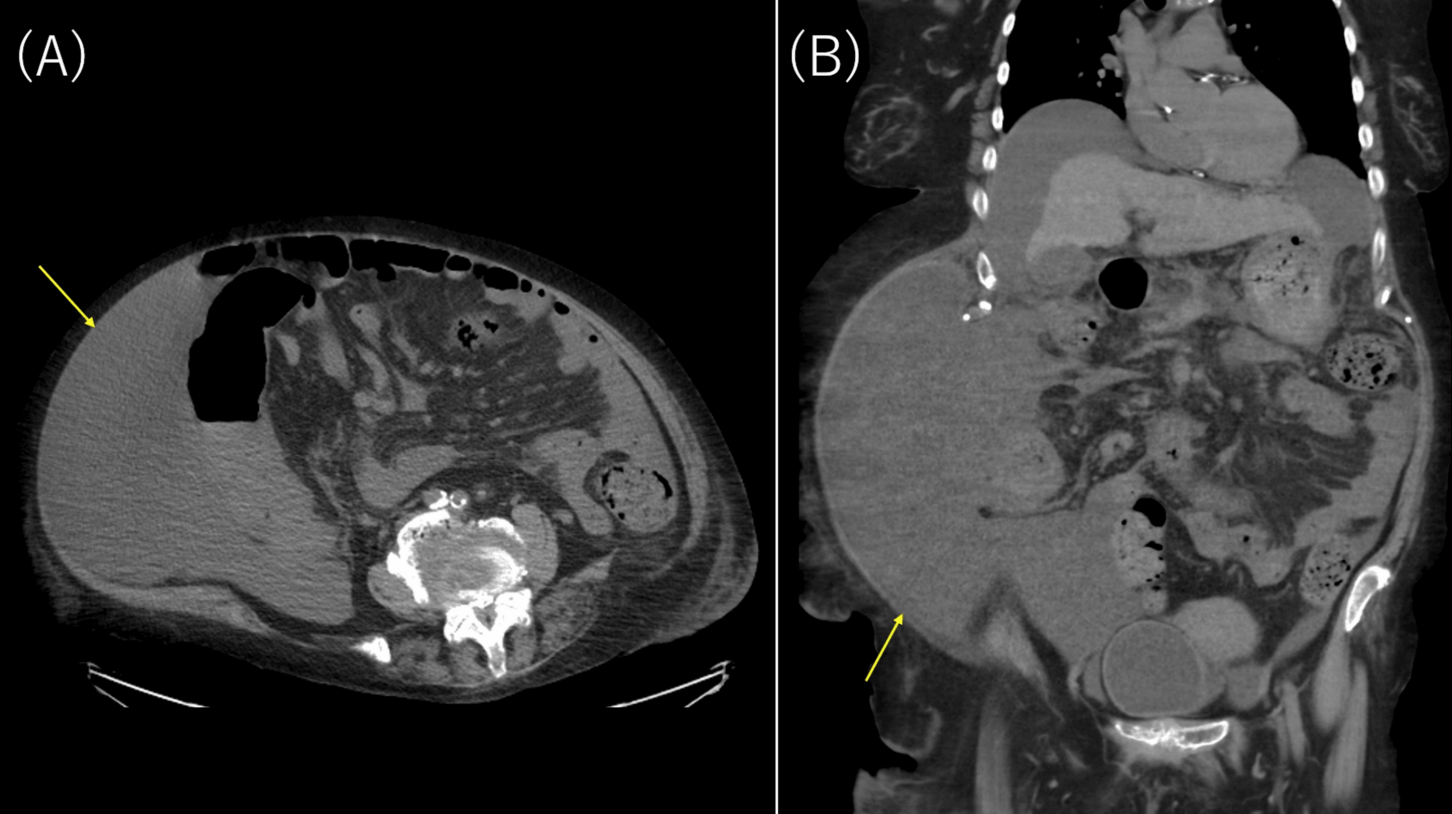
Abdominal CT showing marked ascites (arrows) (A) Axial section, (B) coronal section

Three months after discharge, the patient developed a superficial skin defect (<10 cm diameter, partial-thickness) on the left lower leg at a site of pre-existing psoriatic plaque, prompting readmission for wound care and infection prevention. Physical examination revealed a shallow ulceration with minimal exudate, without surrounding erythema or purulence. Blood tests showed mild leukocytosis (9,600/μL), elevated CRP (1.27 mg/dL), and hyperammonemia consistent with worsening hepatic dysfunction. Chest and abdominal computed tomography (CT) demonstrated marked ascites (Figure 1) but no evidence of pulmonary infection or intra-abdominal abscess. Lower-extremity CT or MRI was not performed, as clinical assessment suggested uncomplicated superficial ulceration without signs of deep infection (no fluctuance, crepitus, or systemic toxicity), and the patient's poor functional status and massive ascites made positioning challenging.

Given the patient's history of recent cellulitis, multiple immunocompromising conditions, and the presence of a skin barrier defect, prophylactic intravenous CEZ at 2 g/day was initiated to prevent secondary bacterial infection during wound healing. The patient remained afebrile and without signs of active infection for the first eight days of hospitalization.

**Figure 2:**
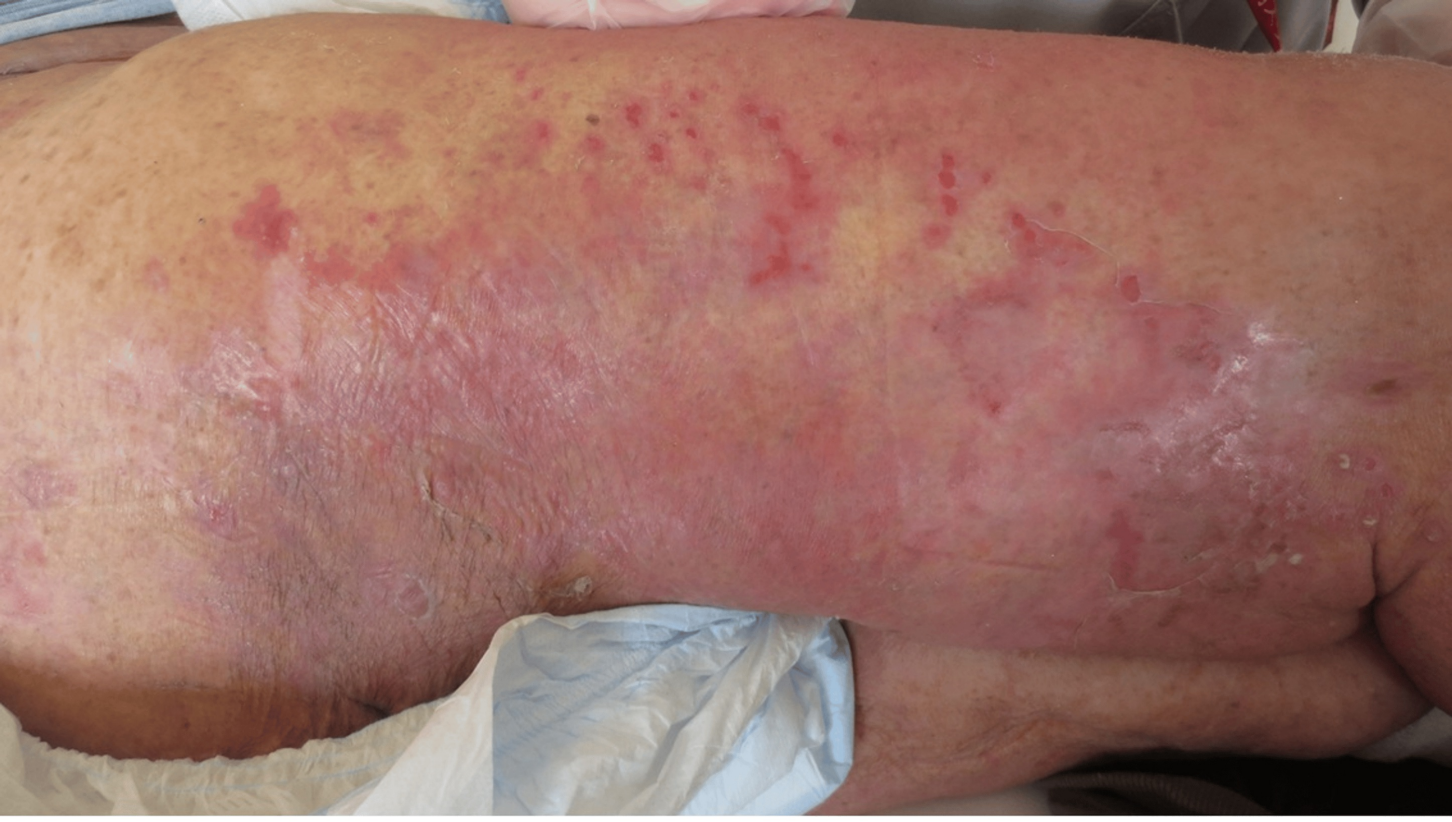
Photograph of the right thigh on day 9 of readmission. Right thigh showing erythema and swelling in the context of hypoalbuminemia from liver cirrhosis. Affected area measured ~20 cm without bullae, necrosis, crepitus, or purpura. Pre-existing psoriatic plaques were visible peripherally but were not involved.

On day 9, the patient developed acute high fever (39.4℃), rigors, and clinical deterioration. Laboratory evaluation revealed marked leukocytosis (13,000/μL) with neutrophilia (88.6%) and elevated CRP (3.55 mg/dL) (Table 1). Physical examination of the skin revealed new-onset sharply demarcated erythema, edema, warmth, and exquisite tenderness on the right thigh, distinct from the left leg ulceration site (Figure 2). The affected area measured approximately 20 cm in diameter, with no bullae, necrosis, crepitus, or purpuric changes. Pre-existing psoriatic plaques were present in the periphery of the affected area but were not directly involved. She was diagnosed with right lower limb cellulitis occurring during CEZ prophylaxis. The Laboratory Risk Indicator for Necrotizing Fasciitis (LRINEC) score was calculated as 4 points [17], indicating low probability of necrotizing fasciitis and supporting conservative management with antibiotics rather than surgical intervention.

**Table 1:** Blood test results on day 9 of the readmission

Tests	Patient Values	Reference Ranges
Red blood cell	329×10⁴/μL	435–555×10⁴/μL
White blood cell	13000/μL	3300–8600/μL
Hemoglobin	9.4 g/dL	13.7–16.8 g/dL
Platelet	18.3 ×10⁴/μL	15.8–34.8 ×10⁴/μL
WBC differential		
Neutrophils	88.6%	40.0–70.0%
Lymphocytes	6.6%	25.0–45.0%
Monocytes	4%	2.0–7.0%
Eosinophils	0.6%	1.0–6.0%
Basophils	0.2%	0.0–1.0%
Blood glucose	131 mg/dL	73–109 mg/dL
Total protein	6.1 g/dL	6.6–8.1 g/dL
Albumin	2.4 g/dL	4.1–5.1 g/dL
Blood urea nitrogen	13 mg/dL	8.0–20.0 mg/dL
Creatinine	0.76 mg/dL	0.65–1.07 mg/dL
Sodium	132 mmol/L	138.0–145.0 mmol/L
Potassium	4.6 mmol/L	3.6–4.8 mmol/L
Chloride	99 mmol/L	101.0–108.0 mmol/L
Estimated glomerular filtration rate	57.7 mL/min/1.73m²	>60 mL/min/1.73m²
C-reactive protein	3.55 mg/dL	0.0–0.14 mg/dL

Given the development of cellulitis despite CEZ prophylaxis, there was high suspicion for a resistant or atypical pathogen. Two sets of blood cultures were obtained, and antimicrobial therapy was switched to oral levofloxacin 500 mg daily, providing broader gram-negative coverage. Additionally, intravenous immunoglobulin (IVIG) at 5 g/day was administered for three consecutive days as adjunctive immunomodulatory therapy, given the patient's profound hypogammaglobulinemia (albumin 2.4 g/dL, reflecting severely reduced hepatic synthetic function) and bacteremia risk in the setting of Child-Pugh class C cirrhosis. A superficial wound culture from the left leg ulceration was also obtained.

Blood cultures from both sets grew *A. lwoffii*. Species identification was performed using the MicroScan WalkAway system (Beckman Coulter, Brea, California, United States), which utilizes biochemical testing for species-level identification. Antimicrobial susceptibility testing was also conducted using the MicroScan WalkAway system with the AST-N0 or equivalent panel, following Clinical and Laboratory Standards Institute (CLSI) guidelines [18]. The isolate demonstrated susceptibility to levofloxacin (minimum inhibitory concentration (MIC) <2 μg/mL), piperacillin, ceftazidime, cefepime, meropenem, ciprofloxacin, trimethoprim-sulfamethoxazole, and minocycline, but resistance to gentamicin (MIC >8 μg/mL) (Table 2). Notably, susceptibility to cefazolin was not reported, consistent with the known intrinsic resistance of *Acinetobacter* species to first-generation cephalosporins.

**Table 2:** Antimicrobial susceptibility testing results for Acinetobacter lwoffii R, resistant; S, sensitive; NA, not applicable

Antibiotic	Minimum inhibitory concentration	Susceptibility
Ampicillin	<8	NA
Piperacillin	<16	S
Cefazolin	<8	NA
Cefmetazole	<16	NA
Ceftazidime	<4	S
Cefepime	<8	S
Meropenem	<1	S
Gentamicin	>8	R
Ciprofloxacin	<1	S
Levofloxacin	<2	S
Trimethoprim-sulfamethoxazole	2	S
Minomycin	<4	S

Wound culture from the left leg ulceration grew extended-spectrum beta-lactamase (ESBL), producing *E. coli *and coagulase-negative* Staphylococcus*; however, these organisms were considered contaminants or colonizers rather than invasive pathogens, as they were obtained from a superficial swab of a non-infected chronic wound site, did not correlate with blood culture results, and the clinical presentation (fever, bacteremia, right thigh cellulitis distant from the wound) did not suggest left leg wound infection as the source.

Levofloxacin therapy was continued, targeting the *A. lwoffii *bloodstream infection and right thigh cellulitis. The patient defervesced within 72 hours, and local inflammatory signs (erythema, warmth, tenderness) gradually improved over the subsequent week. CRP decreased to 0.69 mg/dL by day 19 (Table 3). Levofloxacin was discontinued on day 19 after 10 days of therapy. As the patient's general condition stabilized and no signs of recurrence were observed, follow-up blood cultures were not performed after treatment. Ascites management was optimized with diuretic adjustments, and she was discharged home on day 40 with resolved cellulitis and improved wound healing of the left leg ulceration. Since then, she has been followed up in the outpatient clinic once a month, and no recurrence of cellulitis has been observed.

**Table 3:** The trends in biomarkers during the second hospitalization. After initiation of LVFX on hospital day 9, both fever and inflammatory response improved. LVFX: levofloxacin

Biomarkers	Day 1	Day 9	Day 10	Day 13	Day 19
Body temperature (℃)	37.0	39.4	37.4	37.1	36.9
CRP (mg/dL)	1.27	3.55	9.81	3.95	0.69

## Discussion

This case represents a rare instance of *A. lwoffii* cellulitis and bacteremia in a patient with multiple severe immunocompromising conditions. The convergence of decompensated Child-Pugh class C cirrhosis, diabetes mellitus, and chronic plaque psoriasis created a profound state of immune dysregulation and impaired skin barrier function, predisposing the patient to infection with this typically low-virulence organism.

*A. lwoffii* is a gram-negative coccobacillus that is part of normal human skin flora but possesses relatively low intrinsic virulence compared to more pathogenic *Acinetobacter *species such as* A. baumannii *[13,19]. Unlike* A. baumannii*, which expresses multiple virulence factors, including capsular polysaccharides, biofilm formation capacity, and extensive antimicrobial resistance mechanisms, *A. lwoffii* lacks many of these traits [20]. Its success as a pathogen relies primarily on host susceptibility rather than microbial virulence. In immunocompromised patients, *A. lwoffii* is most commonly associated with catheter-related bacteremia, particularly in those with central venous catheters [13]. Cellulitis caused by *A. lwoffii *is exceedingly rare; a PubMed search revealed fewer than 10 published cases, predominantly occurring in patients with hematologic malignancies undergoing chemotherapy or patients with end-stage renal disease on peritoneal dialysis, with most cases responding favorably to fluoroquinolone or carbapenem therapy [21-26].

In our patient, the infection was likely endogenous in origin, arising from the patient's own cutaneous flora. Endogenous origin was considered most probable given the absence of intravascular catheters, lack of recent hospitalization prior to the second admission, and no identified environmental exposure or nosocomial source. The organism's known colonization of normal human skin, combined with the patient's widespread psoriatic plaques with fissuring and barrier disruption, provided plausible entry sites for translocation from colonization to invasive infection. In states of severe immunocompromise, such translocation from colonization sites to deeper tissues can occur, especially when skin integrity is compromised and local vascular supply is reduced [27].

While cellulitis in healthy individuals is primarily caused by gram-positive bacteria such as *S. pyogenes *and *S. aureus*, immunocompromised patients demonstrate a distinctly different microbial spectrum. Carey and Dall reported that 33% of cellulitis cases in immunocompromised patients were caused by gram-negative bacteria, reflecting altered immune defenses and increased susceptibility to opportunistic pathogens [3]. In cirrhotic patients specifically, gram-negative cellulitis is well-documented and associated with significantly higher mortality rates. Corredoira et al. described gram-negative bacillary cellulitis in cirrhotic patients, noting mortality rates of 30-56% when bacteremia was present [6]. This underscores the critical importance of considering atypical gram-negative organisms in cellulitis management for this vulnerable population, as empiric therapy targeting only gram-positive pathogens may be inadequate.

The successful treatment of our patient with levofloxacin reflects the favorable antimicrobial susceptibility profile typically demonstrated by *A. lwoffii*. Fluoroquinolones provide excellent tissue penetration and maintain good activity against most *A. lwoffii* isolates. The failure of prophylactic cefazolin to prevent *A. lwoffii* cellulitis is not surprising and highlights an important teaching point. *Acinetobacter *species, including *A. lwoffii*, demonstrate intrinsic resistance to first-generation cephalosporins and aminopenicillins due to constitutive production of chromosomal AmpC β-lactamases and reduced outer-membrane permeability [20,28,29]. This case illustrates the limitations of narrow-spectrum prophylactic strategies in severely immunocompromised patients and emphasizes the necessity for culture-directed therapy when unusual or resistant pathogens are suspected.

The addition of intravenous immunoglobulin (IVIG) as adjunctive therapy in our patient was based on her profound immunocompromised state, particularly the severe hypogammaglobulinemia secondary to hepatic synthetic dysfunction (albumin 2.4 g/dL), and the development of gram-negative bacteremia despite ongoing antibiotic prophylaxis. IVIG can theoretically provide passive immunity through pathogen-specific antibodies, enhance opsonization and phagocytosis, and modulate inflammatory responses [30]. While high-quality evidence specifically supporting IVIG use for *A. lwoffii* infections is lacking, limited case reports and small studies have suggested potential benefit in severely immunodeficient septic patients, particularly those with hypogammaglobulinemia [31]. Given our patient's clinical severity (high fever, bacteremia, progressive cellulitis) and inability to mount adequate immune responses, IVIG was considered a reasonable adjunctive measure, though its independent contribution to clinical improvement cannot be definitively determined from this single case.

This case emphasizes several important clinical principles that are particularly relevant for dermatologists managing cellulitis in complex patients. First, clinicians should maintain high suspicion for atypical gram-negative pathogens in patients with multiple immunocompromising conditions. The combination of hepatic cirrhosis, diabetes, and psoriasis creates a particularly high-risk scenario for opportunistic infections with organisms that would rarely cause disease in immunocompetent hosts. Second, prophylactic antibiotic strategies using narrow-spectrum agents (e.g., first-generation cephalosporins) may be insufficient in severely immunocompromised patients and may select for resistant or intrinsically non-susceptible organisms. Third, blood cultures remain crucial in immunocompromised patients with cellulitis, as they may reveal unexpected pathogens that would not be covered by empirical therapy targeting typical skin flora. The identification of *A. lwoffii* bacteremia in our patient was essential for appropriate antimicrobial selection and prognostic assessment. Finally, adjunctive therapies such as IVIG may have a role in managing severe infections in profoundly immunodeficient patients with hypogammaglobulinemia, though evidence remains limited and further study is needed.

These principles are particularly relevant for dermatologists managing cellulitis in patients with chronic inflammatory skin diseases such as psoriasis, where both disease-related barrier dysfunction and potential treatment-related immunosuppression (particularly with systemic agents or biologics) may predispose to atypical gram-negative organisms. When cellulitis develops over or adjacent to psoriatic plaques, or in patients with other barrier-compromised dermatoses (e.g., chronic eczema, chronic leg ulcers), broadened microbiologic consideration and culture-directed antimicrobial therapy may be warranted, particularly if typical empiric therapy fails.

## Conclusions

This case represents one of the few documented instances of *A. lwoffii* cellulitis and bacteremia in a patient with the combined triad of decompensated cirrhosis, diabetes mellitus, and psoriasis vulgaris, a unique constellation of immune-dysregulating conditions that created profound susceptibility to this typically low-virulence organism. The convergence of hepatic immune dysfunction (complement deficiency, impaired neutrophil function, hypogammaglobulinemia), diabetic microangiopathy, hyperglycemia-related immune impairment, and psoriatic skin barrier disruption enabled endogenous skin flora to invade deeper tissues and cause invasive disease. The failure of cefazolin prophylaxis and the subsequent favorable response to levofloxacin underscore the importance of understanding intrinsic antimicrobial resistance patterns in *Acinetobacter* species and the value of culture-directed therapy.

Clinicians managing cellulitis in severely immunocompromised or barrier-compromised patients should maintain heightened suspicion for atypical gram-negative pathogens beyond the typical *Streptococcus* and *Staphylococcus* species. Blood cultures should be routinely obtained in such patients to guide antimicrobial selection, as empiric narrow-spectrum therapy may be inadequate. Further case reporting and prospective microbiologic surveillance of* A. lwoffii *in skin and soft tissue infections may help delineate antimicrobial susceptibility trends, define optimal treatment duration in immunocompromised hosts, and guide empiric therapy strategies for this vulnerable population.
